# Postprandial serum phosphorus and calcium concentrations in adults and children with X-linked hypophosphatemia during burosumab treatment

**DOI:** 10.1093/jbmrpl/ziag008

**Published:** 2026-01-20

**Authors:** Anthony A Portale, Suzanne M Jan de Beur, Craig F Munns, Eric Peng, Marc Vincent, Heather M Heerssen, Erik A Imel

**Affiliations:** Division of Pediatric Nephrology, University of California, San Francisco, San Francisco, CA 94158, United States; Division of Endocrinology and Metabolism, University of Virginia, Charlottesville, VA 22908, United States; Child Health Research Centre, The University of Queensland, Brisbane, QLD 4072, Australia; Department of Endocrinology and Diabetes, Queensland Children’s Hospital, South Brisbane, QLD 4101, Australia; Bioanalytical Development & Biomarker Strategy, Ultragenyx Pharmaceutical, Inc., Novato, CA 94949, United States; Kyowa Kirin, Inc., Princeton, NJ 08540, United States; Kyowa Kirin, Inc., Princeton, NJ 08540, United States; Department of Medicine and Pediatrics, Indiana University School of Medicine, Indianapolis, IN 46202, United States

**Keywords:** cell/tissue signaling: endocrine pathways, PTH/Vit D/FGF23, clinical trials, disease and disorders of/related to bone, osteomalacia and rickets, disorders of calcium/phosphate metabolism

## Abstract

X-linked hypophosphatemia (XLH) is caused by *PHEX* gene variants that result in increased circulating levels of fibroblast growth factor 23 (FGF23). FGF23 in turn decreases renal reabsorption of phosphate and suppresses renal production of 1,25(OH)_2_D, leading to rickets and growth impairment in children and osteomalacia in children and adults. Burosumab is a fully human FGF23-blocking monoclonal antibody approved for treating XLH. Limited data are available on the impact of phosphorus-containing meals or supplements or diurnal variation on serum phosphorus levels, with increases observed in some, but not all studies. It is recommended that serum phosphorus be measured in the morning fasted state when monitoring treatment in patients with XLH. The present substudy of the pivotal pediatric and adult phase 3 clinical trials of burosumab examined the impact of meal consumption and timing around meals on serum phosphorus and calcium levels in children and adults with XLH during burosumab treatment. Thirty-nine participants (pediatric, *n* = 13; adult, *n* = 26) were included. The mean (SD) duration of burosumab treatment prior to the substudy was 15.4 (6.6) mo for pediatric and 24.2 (3.7) mo for adult participants. Serum phosphorus and calcium levels were measured before and after breakfast in children, and before and after both breakfast and lunch in adults. In both age groups, there was no clinically meaningful difference in mean levels of serum phosphorus measured at 1 and 2 h after meals compared to fasted levels, and serum calcium levels remained within the normal range for all pediatric participants and most adults, although interpatient variation was observed. These results suggest that, when fasting is not possible, nonfasting serum phosphorus levels may be a suitable alternative in patients with XLH receiving a stable dose of burosumab.

## Graphical Abstract

**Graphical Abstract:**
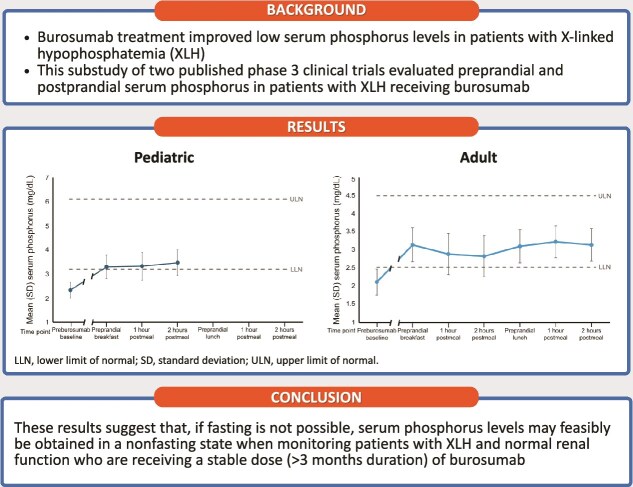

## Introduction

X-linked hypophosphatemia (XLH) is a renal phosphate wasting disorder caused by loss-of-function variants in the phosphate-regulating endopeptidase homolog X-linked (*PHEX*) gene.^1–3^ In the absence of functional *PHEX*, expression and secretion of intact fibroblast growth factor 23 (FGF23) by osteocytes is greatly increased.^4–6^ FGF23 plays a central role in phosphate homeostasis by reducing renal tubular reabsorption of phosphate^1^^,^^7^ and suppressing production of 1,25(OH)_2_D.^8^ In patients with XLH, excess circulating FGF23 thus results in chronic hypophosphatemia, leading to a range of symptoms, including rickets, leg deformities, and impaired growth in children, and osteomalacia, fractures, and pseudofractures in adults.^2^^,^^3^^,^^9^

A major objective of treatment for patients with XLH is to prevent or ameliorate rickets and improve osteomalacia.^2^^,^^3^^,^^10^^,^^11^ Historically, conventional therapy consisted of oral phosphate salts and active vitamin D, which confers risks of nephrocalcinosis and hyperparathyroidism.^3^ Burosumab, a fully human monoclonal antibody against FGF23, is approved for treatment of XLH in children 6 mo of age and older and adults.^12^^,^^13^ By blocking the action of FGF23, burosumab results in increased renal reabsorption of phosphate and production of 1,25(OH)_2_D.^14–16^ In a phase 3 clinical trial in children with XLH who were treated with burosumab for 64 wk (NCT02915705), mean morning fasting serum phosphorus concentrations were higher and improvement in rickets severity and growth were greater than in those receiving oral phosphate and active vitamin D.^17^ In a randomized phase 3 trial in adults with XLH (NCT02526160), a significantly greater percentage of patients receiving burosumab achieved serum phosphorus concentrations above the lower limit of normal (LLN; 2.5 mg/dL [0.81 mmol/L]), measured at 2 or 4 wk postdose averaged across 24 wk (94.1% and 67.6%, respectively), than did those receiving placebo (7.6% and 6.1%, respectively).^18^ Improved phosphorus homeostasis was sustained during subsequent open-label burosumab continuation periods lasting to 48 wk^19^ and 96 wk^20^ of treatment.

In the burosumab clinical trials, serum phosphorus concentration was measured in the morning fasted state to minimize potential effects of phosphorus intake or diurnal variation on serum phosphorus concentration.^17^^,^^18^ Serum phosphorus concentration is known to exhibit periodic variations throughout a 24-h day.^21^^,^^22^ In healthy men ingesting a normal amount of phosphorus, serum phosphorus concentration exhibits a circadian rhythm, characterized by a nadir in late morning, a minor increase to a plateau in the afternoon, and a major peak just after midnight.^21^ Increasing or restricting dietary phosphorus intake altered the time course and magnitude of the peak in serum phosphorus levels.^21^ In healthy volunteers, the quantity and type of phosphorus ingested (ie, inorganic vs organic) also can impact serum phosphorus levels, with the levels being higher after consumption of meals containing high amounts of inorganic phosphorus from food additives (eg, pH control agents, coagulants, and stabilizers) than with meals containing high amounts of organic phosphorus from natural food sources.^23^^,^^24^ Given the potential for the phosphorus content of meals and diurnal variation to affect serum phosphorus concentrations,^21^^,^^23^ it is recommended that, for clinical monitoring of therapy in patients with XLH, serum phosphorus be measured in the morning fasted state.^3^ However, the effect of meal consumption or time of day on serum phosphorus concentrations in burosumab-treated patients remains unknown. In the present study, we examined the effect of meal consumption and time of day on serum phosphorus and calcium concentrations in patients with XLH receiving stable doses of burosumab.

## Materials and methods

Study design and participant eligibility for the phase 3 clinical trials in children and adults with XLH were described previously.^17^^,^^18^ Briefly, in the pediatric clinical trial (NCT02915705), 61 children aged 1-12 yr with XLH who previously received oral phosphate and active vitamin D were randomized (1:1) to receive either burosumab starting at 0.8 mg/kg every 2 wk (titrated based on serum phosphorus concentrations), or oral phosphate and active vitamin D for 64 wk, after which all participants received burosumab alone.^17^^,^^25^ Enrollment criteria included a fasting serum phosphorus concentration <3.0 mg/dL (1.0 mmol/L), a total Thacher rickets severity score of ≥2.0,^17^^,^^26^ and a history of prior treatment with oral phosphate and vitamin D for ≥12 mo for children ≥3 yr or for ≥6 mo for age <3 yr.^17^

In the adult clinical trial (NCT02526160), 134 adults aged 18-65 yr with XLH were randomized (1:1) to receive burosumab starting at 1.0 mg/kg every 4 wk or placebo.^18^ After 24 wk, participants received open-label burosumab at an initial dose of 1.0 mg/kg every 4 wk for 96 wk; a dose reduction was required in some participants due to elevated fasting serum phosphorus concentration.^18–20^ For US study sites, there was an additional open-label extension period of up to 53 wk, for a total treatment duration of up to 149 wk. Enrollment criteria included a fasting serum phosphorus below the LLN, a ratio of renal tubular maximum reabsorption rate of phosphate to glomerular filtration rate (TmP/GFR) of <2.5 mg/dL, corrected serum calcium level <10.8 mg/dL (2.7 mmol/L), and a Brief Pain Inventory worst pain score of ≥4.^18^

For the postprandial substudy, additional inclusion criteria for children were receipt of ≥4 doses of burosumab (≥8 wk of treatment) with at least 80% compliance. Any adult currently treated with burosumab was eligible to be included. All participants, or their caregivers, provided informed consent via the clinical trial consent form, which included the option of a postprandial substudy.

**Figure 1:**
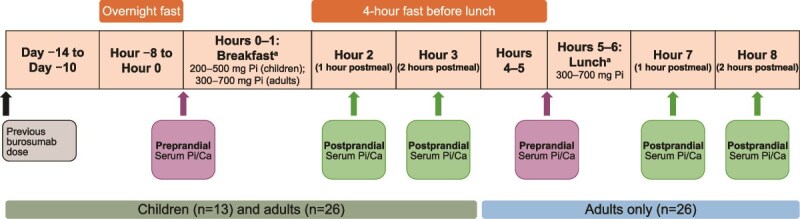
Postprandial substudy design. Ca, calcium; Pi, phosphorus. ^a^Participants were given 1 h to complete meals.

For the substudy participants, baseline serum phosphorus and calcium values were obtained at initiation of the clinical trials, before any dose of burosumab. Pre- and postprandial serum phosphorus and calcium levels were then determined at a single study visit 10-14 d after a burosumab dose (Figure 1). Thus, children were assessed near the end of their dose cycle, while adults were assessed near the middle of their dose cycle. After an overnight fast of at least 8 h, specimens for determination of serum phosphorus and calcium levels were collected before breakfast, and at 1 and 2 h after meal completion. The breakfast meal was representative of a typical Western diet for children and adults, with predefined ranges of phosphorus (approximately 200-500 mg and 300-700 mg, respectively), calories, and carbohydrates. Four hours after completion of the breakfast meal, adult participants received a lunch meal containing 300-700 mg of phosphorus. Blood specimens were again obtained before, and at 1 and 2 h after lunch.

### Statistical analysis

For all substudy participants, changes from morning fasting values in postprandial serum phosphorus concentrations were determined for each participant; unadjusted estimates, CI, and *p* values were calculated using a generalized estimation equation (GEE) model with an exchangeable correlation structure. Due to the small sample size, the GEE model was not adjusted for any confounding factors or covariates.

## Results

Thirteen participants from the pediatric trial and 26 from the adult trial were included in the postprandial substudy (Table 1). Substudy participants were from North America, Europe, or Australia, and the majority were female. The mean (SD; range) duration of prior burosumab treatment in the adults was 24.2 (3.7; 18.1-30.6) mo and, in the children, 15.4 (6.6; 2.3-22.1) mo.

**Table 1:** Baseline characteristics in substudy patients.

Characteristic	Pediatric study (*N* = 13)	Adult study (*N* = 26)
**Age, yr**		
**Mean (SD)**	6.6 (3.0)	42.7 (11.8)
**Min, max**	2.9, 12.8	23.9, 64.8
**Sex, *n* (%)**		
**Female**	10 (76.9)	20 (76.9)
**Male**	3 (23.1)	6 (23.1)
**Race, *n* (%)**		
**White**	12 (92.3)	23 (88.5)
**Asian**	0	1 (3.8)
**Black or African American**	0	1 (3.8)
**Other**	1 (7.7)	1 (3.8)
**Ethnicity, *n* (%)**		
**Hispanic or Latino**	5 (38.5)	3 (11.5)
**Not Hispanic or Latino**	8 (61.5)	23 (88.5)
**Height *z* score**		
**Mean (SD)**	−2.27 (1.06)	NR
**Median (range)**	−2.02 (−4.9, −1.2)	NR
**Laboratory test before starting burosumab**		
**Fasting serum calcium (mg/dL), mean (SD)**	9.4 (0.2)	9.1 (0.4)
**Fasting serum phosphorus (mg/dL)**		
**Mean (SD)**	2.4 (0.3)	2.1 (0.4)
**Median (range)**	2.4 (1.8-2.8)	2.1 (1.5-3.0)
**Fasting serum phosphorus (mmol/L)**^**a**^		
**Mean (SD)**	0.8 (0.1)	0.7 (0.1)
**Median (range)**	0.8 (0.6-0.9)	0.7 (0.5-1.0)
**TmP/GFR (mg/dL), mean (SD)**	2.1 (0.4)^b^	1.7 (0.5)
**Alkaline phosphatase, U/L, mean (SD)**	499 (140)	NR
**Serum 1,25(OH)**_**2**_**D, pg/mL, mean (SD)**	51 (21)^c^	29 (15)
**Baseline preprandial substudy assessments**		
**Duration of prior burosumab treatment at time of the substudy (mo), mean (range)**	15.4 (2.3, 22.1)	24.2 (18.1, 30.6)
**Time since last dose of burosumab (d)**		
**Mean (SD)**	13.6 (1.9)	13.0 (1.8)
**Median (range)**	14.0 (10.0-17.0)	13.5 (9.0-16.0)
**Morning fasting laboratory tests**		
** Fasting serum calcium (mg/dL), mean (SD)**	9.6 (0.4)	8.9 (0.4)
** Fasting serum calcium (mmol/dL), mean (SD)**^**d**^	2.4 (0.1)	2.2 (0.1)
** Fasting serum phosphorus (mg/dL)**		
** Mean (SD)**	3.3 (0.5)	3.1 (0.5)
** Median (range)**	3.3 (2.3-4.0)	3.1 (2.4-4.1)
** Fasting serum phosphorus (mmol/L)** ^**a**^		
** Mean (SD)**	1.1 (0.2)	1.0 (0.2)
** Median (range)**	1.1 (0.7-1.3)	1.0 (0.8-1.3)

Abbreviations: NR, not reported; TmP/GFR, ratio of renal tubular maximum phosphate reabsorption rate to glomerular filtration rate.

a Phosphorus conversion factor of 0.323 for mg/dL to mmol/L conversion.^27^

b Data for 2 patients was missing.

c Data for 1 patient was missing.

d Total calcium conversion factor of 0.250 for mg/dL to mmol/L conversion.^27^

**Figure 2:**
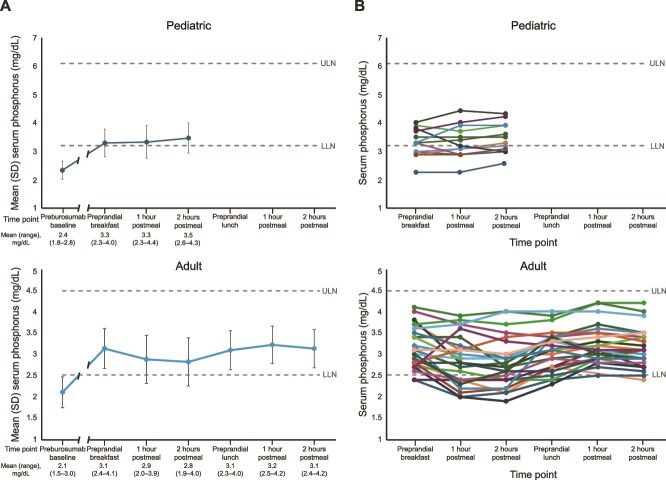
(A) Mean (SD) and (B) individual participant pre- and postprandial serum phosphorus levels in children^a^ and adults^b^. LLN, lower limit of normal; ULN, upper limit of normal. ^a^Value is missing for one pediatric patient at the 2-h postmeal timepoint. ^b^Values are missing for one adult patient at the 2-h postbreakfast timepoint and for one adult at the 1-h postlunch timepoint.

For the pediatric substudy participants, the baseline mean (SD) morning fasting serum phosphorus concentration, obtained before exposure to burosumab, was 2.4 (0.3) mg/dL (0.8 [0.1] mmol/L), a value below the LLN (defined as 3.2 mg/dL [1.0 mmol/L]) Figure 2A). When measured at the baseline of the postprandial substudy, which occurred 10-17 d after the prior burosumab dose, the morning fasting serum phosphorus level was increased to 3.3 (0.5) mg/dL (1.1 [0.2] mmol/L), with individual values being above the LLN in 8 of the 13 children (Figure 2). At 1 and 2 h after breakfast, mean serum phosphorus levels were unchanged from fasting levels, being 3.3 (0.6) mg/dL (1.1 [0.2] mmol/L) and 3.5 (0.5) mg/dL (1.1 [0.2] mmol/L) at 1 and 2 h postprandially, respectively (Table 2, Figure 2A). Individual variation in postprandial serum phosphorus levels was observed among the participants; however, individual postprandial phosphorus values were within ±0.5 mg/dL of their fasting level in 11 (85%) of the 13 children (Figure 2B). In one child, postprandial serum phosphorus was lower than the fasting level by 0.8 mg/dL (3.8-3.0 mg/dL), and, in one child, the postprandial value was higher than the fasting level by 0.6 mg/dL (3.3-3.9 mg/dL). In no participant did serum phosphorus levels exceed the upper limit of normal for age (ULN defined as 6.1 mg/dL [2.0 mmol/L]).

**Table 2:** Comparison of postprandial vs morning fasting serum phosphorus in children with XLH utilizing a GEE model^a^ (*N* = 13).

Assessment time vs morning fasting	Mean change in sPi, mg/dL: unadjusted estimate (CI)	*p-*value
**Postbreakfast**		
**1 h**	0.03 (−0.14, 0.20)	.718
**2 h**	0.15 (−0.04, 0.34)	.125

Abbreviations: CI, 95% confidence interval; GEE, generalized estimation equation; sPi, serum phosphorus; XLH, X-linked hypophosphatemia.

a GEE model with an exchangeable correlation structure. The model was not adjusted for any confounding factors or covariates due to the limitation of the sample size.

In the adult participants, the baseline (before any burosumab) mean morning fasting serum phosphorus level was 2.1 (0.4) mg/dL (0.7 [0.1] mmol/L), below the LLN (defined as 2.5 mg/dL [0.8 mmol/L]) (Figure 2A). When measured at the baseline of the postprandial substudy, which occurred 9-13 d after the prior dose of burosumab, the mean morning fasting phosphorus level was 3.1 (0.5) mg/dL (1.0 [0.2] mmol/L), with individual values being above the LLN in 24 (92%) of 26 participants. At 1 and 2 h after breakfast, phosphorus values trended downward slightly, with mean values being lower by 0.3 mg/dL (*p* < .001) (Table 3, Figure 2). Phosphorus values then trended upward, and mean values before and at 1 and 2 h after lunch were not different from morning fasting levels.

**Table 3:** Comparison of postprandial vs morning fasting serum phosphorus in adults with XLH utilizing a GEE model^a^ (*N* = 26).

Assessment time vs morning fasting	Mean change in sPi, mg/dL: unadjusted estimate (CI)	*p* value
**Postbreakfast**		
**1 h**	−0.27 (−0.42, −0.11)	<.001
**2 h**	−0.32 (−0.48, −0.15)	<.001
**Prelunch**	−0.04 (−0.19, 0.11)	.611
**Postlunch**		
**1 h**	0.07 (−0.07, 0.22)	.317
**2 h**	0 (−0.14, 0.13)	.956

Abbreviations: CI, 95% confidence interval; GEE, generalized estimation equation; sPi, serum phosphorus.

a GEE model with an exchangeable correlation structure. The model was not adjusted for any confounding factors or covariates due to the limitation of the sample size.

Individual variation in postprandial phosphorus levels was observed in the adult participants (Figure 2B). The number of participants whose serum phosphorus levels were within the normal range was 24 (92%) in the morning fasting state, 16 (62%) postbreakfast, 23 (88%) before lunch, and 25 (96%) postlunch. Individual postprandial serum phosphorus values were within ±0.5 mg/dL of the morning fasting levels in 15 (58%) adults postbreakfast, 22 (85%) adults before lunch, and 19 (73%) adults postlunch. In no adult did individual postprandial values exceed the ULN (defined as 4.5 mg/dL [1.5 mmol/L] in adults).

**Figure 3:**
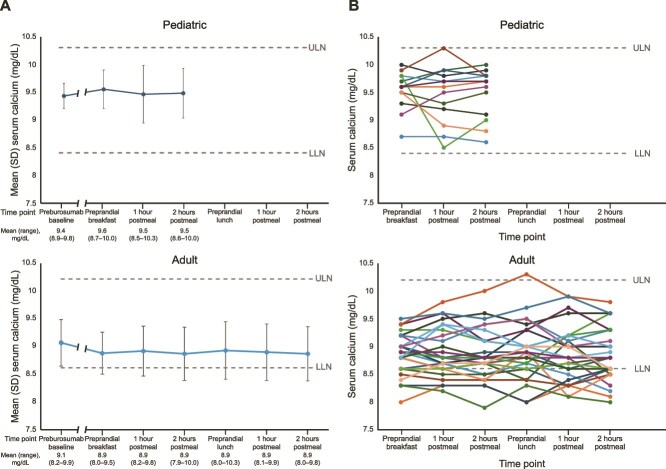
(A) Mean (SD) and (B) individual participant pre- and postprandial serum calcium levels in children and adults. LLN, lower limit of normal; ULN, upper limit of normal.

In both children and adult participants, mean (SD) morning fasting serum calcium levels were within the normal range (Figure 3A and B). During the postprandial study period, serum calcium levels showed wide individual variation in both participant groups, but values remained within the normal range for all pediatric participants (ages 2-18 yr: 8.4-10.3 mg/dL) and most adults (8.6-10.2 mg/dL), with one prelunch value exceeding the ULN in the adult group. For 11/13 (85%) children postbreakfast and 24/26 (92%) adults postbreakfast and before lunch, postprandial serum calcium remained within ±0.5 mg/dL of the fasting level.

## Discussion

Literature regarding the effects of meals and time of day on serum phosphorus concentration is limited.^23^^,^^24^^,^^28^ The majority of studies were conducted in patients with CKD or in healthy volunteers with small sample sizes and differing amounts and types of phosphorus formulations administered.^23^^,^^24^^,^^28^ To the best of our knowledge, the present report is the first to examine the effect of meals and time of day on serum phosphorus levels during treatment of patients with XLH with burosumab.

We observed that, in children and adults with XLH who had received burosumab for a minimum of 10 wk, mean serum phosphorus levels measured after breakfast in children and after lunch in adults differed little from those measured in the morning fasting state. Additionally, most patients’ nonfasting values were within ±0.5 mg/dL of their morning fasting level and no participant had an increase in serum phosphorus above the ULN. Thus, serum phosphorus levels changed little after intake of meals containing organic phosphorus estimated at 200-500 mg for the children and 300-700 mg for the adults. These findings are consistent with those of Kawamura et al., who found that, in 6 healthy volunteers, consumption of a meal high in organic phosphorus (1200 mg) did not significantly increase serum phosphorus levels when measured 30, 60, and 120 min postprandially.^23^ Similarly, Nishida et al. found that, in 8 healthy volunteers, ingestion of meals containing 400 mg of organic phosphorus did not increase serum phosphorus levels significantly from 30 min to 8 h postprandially.^24^ However, when phosphorus intake was increased to 800 or 1200 mg by the addition of readily absorbed neutral phosphate salts, postprandial serum phosphorus levels did increase.^24^ In the current study, the meal content of phosphorus (ie, 200-500 mg for children and 300-700 mg for adults) is within the range of phosphorus ingested in the studies of Nishida et al. and Kawamura et al.^23^^,^^24^

In the pediatric participants, pre- and postbreakfast values were comparable to fasting levels, although individual variability was observed. Following GEE analysis, 1- and 2-h postbreakfast levels were not significantly different than fasting values.

When individual phosphorus values were assessed in the adult participants, a slight downward trend in postbreakfast serum phosphorus levels was observed in more than half of the patients. Upon GEE analysis, 1- and 2-h postbreakfast levels were slightly but significantly lower than fasting values; however, such a change is unlikely to be of clinical significance. Pre- and postlunch values were similar to fasting levels with much less individual variability and, per GEE analysis, were not significantly higher than fasting morning values.

Mean postprandial concentrations of calcium were stable, with individual levels remaining below the ULN in all the pediatric participants and all but one adult participant just before lunch. These observations are comparable to those observed in prior studies of healthy volunteers in which, regardless of source or quantity of dietary phosphorus, little fluctuation occurred in postprandial levels of serum calcium.^23^^,^^24^

Currently, the US product label for burosumab recommends that, in children with XLH, serum phosphorus levels be monitored in the fasting state at 4 wk intervals for the first 3 mo after initiating burosumab, 4 wk after a dose adjustment, and thereafter as appropriate.^29^ Similar guidance to monitor fasting serum phosphorus is given for adults with XLH receiving burosumab, with the additional recommendation to measure fasted serum phosphorus 2 wk postdose and 2 wk after a dose adjustment.^29^ Recently published EU clinical practice recommendations for XLH note that obtaining blood and urine specimens in infants and children in the fasting state is often impractical.^3^ Importantly, any measurement of serum phosphorus must consider its timing in terms of duration since the prior burosumab dose for interpretation. The results of the current study demonstrate that mean serum phosphorus levels measured 1 and 2 h after breakfast in children and adults, and before and 1 and 2 h after lunch in adults, on average, are little different than morning fasting levels. Similarly, mean serum calcium levels were relatively stable throughout the substudy day. These data suggest that, in both pediatric and adult patients with XLH who are receiving a stable dose of burosumab (ie, >3 mo duration), serum phosphorus levels may be clinically monitored in the nonfasting state when fasting is not possible, provided that renal function is normal and remains unchanged.

Limitations of the current study include a small sample size for both pediatric and adult patients. Additionally, the study participants were in clinical trials on stable doses of burosumab and had normal renal function, which may make it difficult to generalize the results to patients who do not share similar circumstances. In children, serum phosphorus and calcium measurements were not obtained beyond late morning; thus, it is not known whether values obtained in the afternoon would accurately represent those obtained earlier in the day. In addition, we observed substantial interpatient variability in postprandial serum phosphorus and calcium levels. The phosphorus content and macronutrient composition of the meals consumed was not standardized, which may have contributed to the interpatient variability of phosphorus and calcium levels observed.

### Conclusions

In this substudy of children and adults with XLH receiving burosumab, mean serum phosphorus and calcium concentrations, measured 1 and 2 h postprandially, did not go above the age-adjusted upper limits of normal in any pediatric or adult participant. Additionally, calcium concentrations were below the ULN in all pediatric participants and only 1 adult had a prelunch serum calcium value exceeding the ULN. These results suggest that, for ongoing monitoring of patients with XLH and normal renal function receiving stable doses of burosumab, serum phosphorus and calcium concentrations may feasibly be determined in a nonfasted state when fasting is not possible. Further research in a larger and more diverse patient population could potentially increase the generalizability of the observed results.

## Data Availability

The data that support the findings of this study are available from Kyowa Kirin, upon reasonable request.

## Clinical trial registration

NCT02915705 and NCT02526160, available from www.clinicaltrials.gov.
